# Is the Cerebellum the Optimal Reference Region for Intensity Normalization of Perfusion MR Studies in Early Alzheimer’s Disease?

**DOI:** 10.1371/journal.pone.0081548

**Published:** 2013-12-27

**Authors:** María Lacalle-Aurioles, Yasser Alemán-Gómez, Juan Adán Guzmán-De-Villoria, Isabel Cruz-Orduña, Javier Olazarán, José María Mateos-Pérez, María Elena Martino, Manuel Desco

**Affiliations:** 1 Departamento de Bioingeniería e Ingeniería Aeroespacial, Universidad Carlos III de Madrid, Leganés, Madrid, Spain; 2 Instituto de Investigación Sanitaria Gregorio Marañón, Madrid, Spain; 3 Centro de Investigación Biomédica en Red de Salud Mental (CIBERSAM), Madrid, Spain; 4 Servicio de Radiodiagnóstico. Hospital General Universitario Gregorio Marañón, Madrid, Spain; 5 Servicio de Neurología, Hospital General Universitario Gregorio Marañón, Madrid, Spain; Torrey Pines Institute for Molecular Studies, United States of America

## Abstract

The cerebellum is the region most commonly used as a reference when normalizing the intensity of perfusion images acquired using magnetic resonance imaging (MRI) in Alzheimer’s disease (AD) studies. In addition, the cerebellum provides unbiased estimations with nuclear medicine techniques. However, no reports confirm the cerebellum as an optimal reference region in MRI studies or evaluate the consequences of using different normalization regions. In this study, we address the effect of using the cerebellum, whole-brain white matter, and whole-brain cortical gray matter in the normalization of cerebral blood flow (CBF) parametric maps by comparing patients with stable mild cognitive impairment (MCI), patients with AD and healthy controls. According to our results, normalization by whole-brain cortical gray matter enables more sensitive detection of perfusion abnormalities in AD patients and reveals a larger number of affected regions than data normalized by the cerebellum or whole-brain white matter. Therefore, the cerebellum is not the most valid reference region in MRI studies for early stages of AD. After normalization by whole-brain cortical gray matter, we found a significant decrease in CBF in both parietal lobes and an increase in CBF in the right medial temporal lobe. We found no differences in perfusion between patients with stable MCI and healthy controls either before or after normalization.

## Introduction

Alterations in cerebral hemodynamic processes are thought to be involved in the pathogenesis of Alzheimer’s disease (AD) [1], [2], [3]. Furthermore, a correspondence has been demonstrated between decreased cerebral perfusion and neuronal deactivation, purportedly as a consequence of neurovascular coupling [4], [5]. Regional cerebral perfusion is associated with tissue metabolic requirements [6]. This physiological mechanism may underlie the association between perfusion deficits in the parietotemporal regions and rapid cognitive decline in AD patients [7]. Consequently, measurement of cerebral perfusion using functional imaging may provide useful information for early detection and tracking of AD. Classically, this assessment has been carried out using nuclear medicine techniques, such as positron emission tomography (PET) or single-photon emission computed tomography (SPECT). However, MRI was recently proposed as a potentially useful tool for the evaluation of cerebral perfusion in AD patients [8]. Dynamic susceptibility contrast (DSC) MRI is the most widely used perfusion-weighted MRI technique. DSC-MRI also has the advantages of shorter acquisition time, lower cost and no need for ionizing radiation. In addition, in contrast to PET and SPECT, structural and perfusion MRI metrics are easily obtained in the same scan session, thus facilitating clinical routine and patient comfort. However, perfusion MRI is affected by wide physiological variability in perfusion measurements across subjects. This variability, which is due to uncontrolled biological and experimental factors [9], makes it difficult to detect group differences, especially in small samples. In order to achieve more sensitive detection of disease-dependent patterns of altered perfusion, variability can be improved by normalizing data before performing comparative analysis.

The most widely used intensity normalization method computes the ratio of a region of interest (ROI) value to the average perfusion value of all voxels within a reference region.

In nuclear medicine studies, the cerebellum, whole-brain gray matter (GM) and whole-brain white matter (WM) have traditionally been considered good reference regions for intensity normalization [10]. The cerebellum is the most commonly used reference region in SPECT perfusion studies in AD patients, since it has been reported to provide unbiased estimations [11], [12], [13]. The idea that the cerebellum remains unaffected by AD is controversial, since some authors have reported reduced function in this region in late dementia, suggesting that normalization by this region in advanced AD may lead to errors [14]. Using DSC-MRI, other authors recently observed a significant decrease in cerebral blood flow (CBF) in the cerebellum of AD patients [15].

The cerebellum is also the most widely used region for normalization in perfusion-weighted MRI studies of patients with AD [16], [17], [18], although the visual cortex [19], basal ganglia [20] and primary motor cortex [21] have also been used. In MRI studies, no reports confirm that the cerebellum is an optimal reference region for normalization or evaluate the consequences of using different normalization regions in perfusion-weighted MRI measurements.

In this study, we address the effect of using different regions for normalization in an analysis of CBF parametric maps comparing patients with mild cognitive impairment (MCI), AD patients and healthy controls. For this purpose, we normalized CBF maps by whole-brain cortical GM, whole-brain WM and the cerebellum.

## Materials and Methods

### Ethics Statement

Written informed consent for all procedures and examinations of the study was obtained from all participants. In the case of the patients, the study neurologist informed about the study procedures to the patient and the caregiver (next of kin). The study neurologist, who also performed mental status exam, established the capacity/ability of the patient to consent. The ethics committee of Hospital General Universitario Gregorio Marañón approved the study.

### Participants

The study sample comprised 43 patients and 20 controls. Patients were divided into 2 groups: those with stable MCI (n = 15) and those with mild AD (n = 28).

The subjects were recruited prospectively by a senior neurologist with expertise in behavioural neurology during his routine clinical practice in the behavioural neurology clinic of Hospital General Universitario Gregorio Marañón. Potential existence of cognitive impairment was investigated in all the participants by means of a semi-structured interview and a physical and neurological examination. All the participants received a formal battery of neuropsychological tests including the California Verbal Learning Test (CVLT) [22], the Frontal Assessment Battery (FAB) [23], a verbal fluency test [24], Addrenbrooke’s Cognitive Examination (ACE) [25] and the Rey-Osterrieth Complex Figure Test (ROCF) [26]. Routine blood determinations, including complete blood count, glucose, creatinine, hepatic enzymes, sodium, potassium, calcium, thyroid-stimulating hormone, B12 and folate, were obtained in the MCI and AD groups to detect causes of cognitive impairment other than AD. Subjects were excluded if they presented any medical, psychiatric, or neurological condition (except the possibility of AD) that could affect cognition. Patients were not receiving medication for AD at the time of study. General inclusion criteria were age over 60 years and ability to read and write. The specific criteria for inclusion in each of the study groups are detailed in the following paragraphs.

MCI group: MCI was diagnosed using the criteria of Winblad et al. [27], which extend the criteria of Petersen et al. [28]. The MCI criteria included self- and/or informant report of impairment in any cognitive function with preserved basic activities and no (or minimal) impairment in complex instrumental functions. Cognitive impairment had to be supported by an abnormal performance (1–1.5 SD below the expected performance for age and education) in one or more tests from the neuropsychological battery. In addition, MCI patients did not meet the criteria for dementia according to the Diagnostic and Statistical Manual of Mental Disorders (DSM-IV-TR) [29] and were at stage 0.5 in the Clinical Dementia Rating Scale (CDR) [30]. After 2 years of clinical follow-up, the criteria for MCI persisted in this group.

AD group: Probable AD was diagnosed using the criteria established by the National Institute of Neurological and Communicative Disorders and Stroke – Alzheimer Disease and Related Disorders Association (NINCDS – ADRDA) [31]. Hence, deterioration in memory and other cognitive functions had to be documented and instrumental activities of daily living had to be affected. The diagnosis of mild AD was achieved with a score of 1 (i.e., mild dementia) in the CDR [30]. The AD group comprised 12 patients recruited with probable AD and 16 subjects recruited at the stage of MCI (CDR = 0.5) that converted to probable AD after 2 years of follow-up.

Control subjects. Control subjects were chosen from among caregivers attending the behavioral neurology clinic and from among researchers’ acquaintances. These control subjects did not present relevant cognitive complaints. To be included in this study, they had to score above 24 in the MMSE test [32].

### Image Acquisition

MRI data were acquired using a 1.5T system (Philips Medical Systems, Best, The Netherlands). The imaging protocol included a volumetric T1-weighted 3D gradient echo, which was used for tissue segmentation (FA = 30°; TR = 16 ms, TE = 4.6 ms; matrix size = 256×256; FOV = 256×256 mm; and 100 slices with slice thickness = 1.5 mm).

Perfusion-weighted images (PWI) were obtained using an echo-planar imaging sequence (EPI factor = 61, FA = 40°; TR = 1439 ms, TE = 30 ms; matrix size = 128×128; FOV = 230×230 mm; section thickness = 5 mm) after the injection of a bolus of gadolinium chelate (10 ml of Gadobutrol; Gadovist® Bayer-Schering AG, Berlin, Germany). Forty volumes (30 slices each) per subject were obtained during the administration of the gadolinium contrast (automatic injector, 4 ml/s). After the gadolinium bolus, 30 ml of saline solution was administered (4 ml/s).

**Figure 1:**
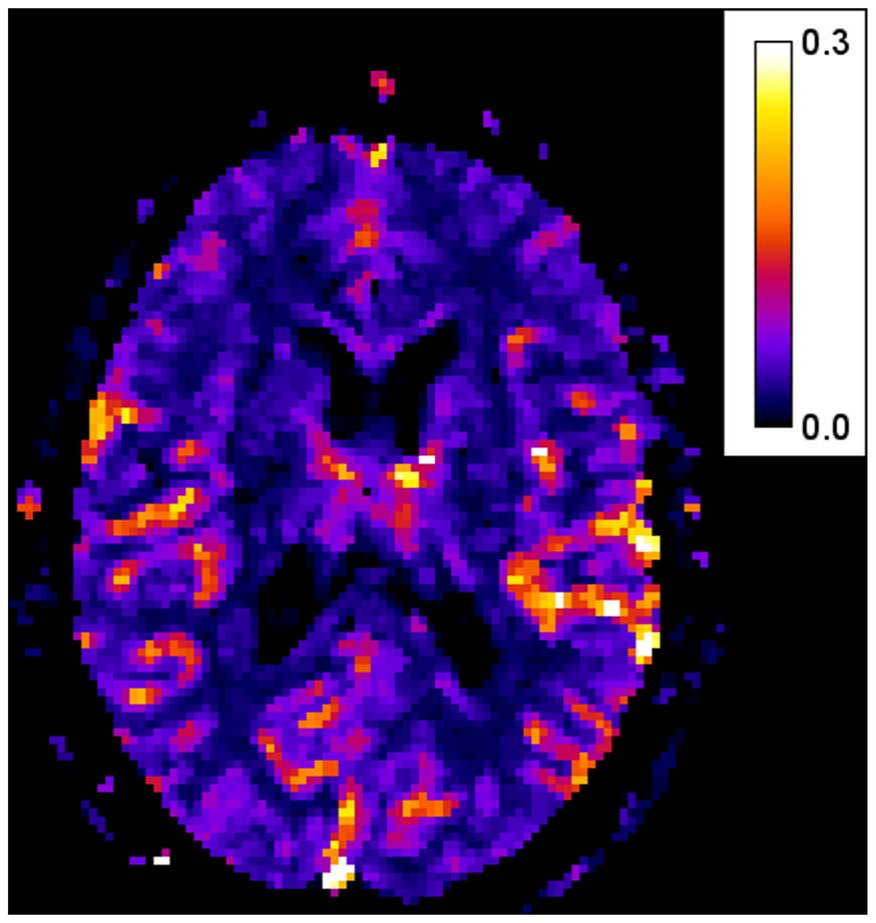
Example of CBF parametric map.

CBF maps (Fig. 1) were obtained according to the following equation:
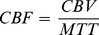



Where
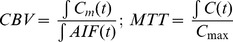



In the previous equation, there are two different concentrations: the concentration that can be measured directly from the image data (C_m_(t)) and the concentration resulting from a deconvolution operation with the arterial input function (AIF). Both concentrations can be expressed mathematically as
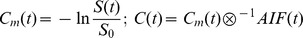



Where S(t) is the MRI image signal, S_0_ is the signal in the first six frames, when the contrast bolus still is not present and 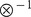 represents the deconvolution operation.

The AIF was calculated automatically using the method detailed in Rempp et al. [33]. In order to remove noise and other undesired second-order effects, the contrast curves where fitted to a gamma function according to the linearization method proposed in Li et al. [34].

### Image Analysis

#### Brain mask extraction

Brain masks were estimated from skull-stripped baseline images generated using the VBM8 toolbox (available at: http://dbm.neuro.uni-jena.de/vbm) for the SPM8 package (Wellcome Trust Centre for Neuroimaging, London, UK; available at: http://www.fil.ion.ucl.ac.uk/spm). This algorithm produces a skull-stripped ‘p0’ image that consists of brain tissue classified into GM, WM and cerebrospinal fluid (CSF). The ‘p0’ images were binarized and visually inspected to correct brain segmentation errors. The skull was removed manually if necessary.

### FreeSurfer Processing

T1-weighted images were processed using the FreeSurfer package (version 4.5.1, http://surfer.nmr.mgh.harvard.edu) to estimate cortical gray matter volume per ROI. Brain mask images obtained from the VBM8 toolbox were introduced into the default FreeSurfer processing pipeline because they provide more accurate skull stripping.

The white and gray cortical surfaces were reconstructed from the raw unaligned images in native space, with the methods described by Fischl and Dale [35] and Dale et al. [36]. The reconstruction process was supervised and corrected when necessary by an operator blind to the subject’s diagnosis. The measurement technique used by FreeSurfer has been validated using histological measurements [37] and manual measurements [38]. Cortical parcellation into gyral-based ROIs was calculated according to the Desikan-Killiany cortical atlas [39]. Whole-brain WM, amygdalae, hippocampi and cerebellum were segmented according to [40]. T1-weighted images were coregistered with the CBF parametric maps using mutual information methods [41]. After coregistration, masks of every gyral-based ROI, whole-brain WM and cerebellum were applied to CBF maps. An average CBF value per ROI was then computed.

Perfusion information for the different cerebral lobes (frontal, parietal, temporal and occipital) was obtained by assigning a set of gyri to each lobe according to the cortical structure described in the Desikan-Killiany atlas [39]. Temporal lobes were divided into medial and lateral regions. Whole-brain cortical GM was calculated using the average of perfusion data for every lobe. Table 1 summarizes the volume size of the three reference region compared in this paper.

**Table 1:** Mean volume (cm^3^) and standard deviation (SD) of the three reference regions for normalization.

	*Controls Mean (SD)*	*MCI Mean (SD)*	*Alzheimer Mean (SD)*
Whole-brain cortical GM	166.59 (17.48)	163.25 (16.53)	148.70 (15.78)
Whole-brain WM	420.56 (51.54)	417.97 (42.95)	388.35 (56.92)
Cerebellum	125.61 (15.01)	124.55 (12.82)	117.23 (10.63)

### Statistical Analysis

We used one-way ANOVA to test the hypothesis that no differences existed in mean CBF values in any of the three chosen reference regions between groups (controls, MCI and AD) in order to rule out any between-group bias in reference regions for normalization. We also used ANOVA to test mean CBF values for differences per lobe between groups before and after normalization by the three reference regions. When the result of ANOVA was significant, we used a post hoc procedure (Dunnett’s test) to compare means between patients groups and controls and we calculated the effect size (Cohen’s coefficient, d) in order to assess the magnitude of those differences. The effect of using different regions for normalization on the intra-group variability of PWI data was assessed through the coefficient of variation (CV). We also estimated the components of variance for the diagnosis group and normalization region to ensure that any reduction in variability after normalization (measured using the CV) was due to a reduction in intra-group variability and not to a reduction in between-group differences. An ANCOVA model including age as covariate was used to test between-group differences in GM volume in the medial temporal cortex, amygdalae and hippocampi. Data were analyzed using Statistical Analysis System (SAS) version 9.0 (SAS Institute Inc., Cary, NC, USA).

## Results

### Demographic Data

Demographic and clinical data of the participants are shown in Table 2. No significant between-group differences were found for any of the variables, except in the MMSE score, which was lower in the AD group than in the MCI and control groups.

**Table 2:** Demographic and clinical data.

	*Controls* *n = 20*	*MCI* *n = 15*	*Alzheimer* *n = 28*
Age in years (SD)	71.65 (7.04)	70.87 (9.71)	74.86 (6.97)
Sex (Female:Male)	11∶9	7∶8	15∶13
Years of education (SD)	9.1 (4.36)	7.07 (3.39)	6.79 (4.07)
MMSE (SD)	27.55 (2.09)	26.20 (1.97)	20.61 (4.08)*

ANOVA of group differences (*p*<0.0001). Significant differences were found between the Alzheimer group and the controls, and between the Alzheimer group and the MCI group. MMSE, Mini Mental State Examination; SD, standard deviation.

### Between-group Bias in Reference Regions

No statistically significant differences were found in mean CBF values between the groups in any of the three reference regions studied. Intra-group variability in CBF intensity values was similar in the three regions: around 40% in the control group and 50% in the MCI and AD groups (Table 3).

**Table 3:** Mean CBF values and intra-group variability in intensity for the three reference regions.

	*Controls n = 20*		*MCI n = 15*		*Alzheimer n = 28*		*ANOVA*		
	Mean (SD)	CV	Mean (SD)	CV	Mean (SD)	CV	*F*	*p*	
CBFcgm	0.11 (0.04)	39.2	0.11 (0.05)	48.8	0.11 (0.05)	49.3	0.03	NS	
CBFcer	0.30 (0.12)	41.0	0.29 (0.14)	50.2	0.28 (0.14)	50.5	0.14	NS	
CBFwm	0.07 (0.03)	41.7	0.07 (0.03)	50.3	0.06 (0.03)	50.1	0.28	NS	

CV, coefficient of variation (%); SD, standard deviation; CBFcgm, cerebral blood flow of whole-brain cortical gray matter; CBFcer, cerebral blood flow of cerebellum; CBFwm, cerebral blood flow of whole-brain white matter; *F*, ANOVA *F* value; *p*, ANOVA *p* value; NS, not significant.

### Effects of Normalization on between-group Analysis

**Figure 2:**
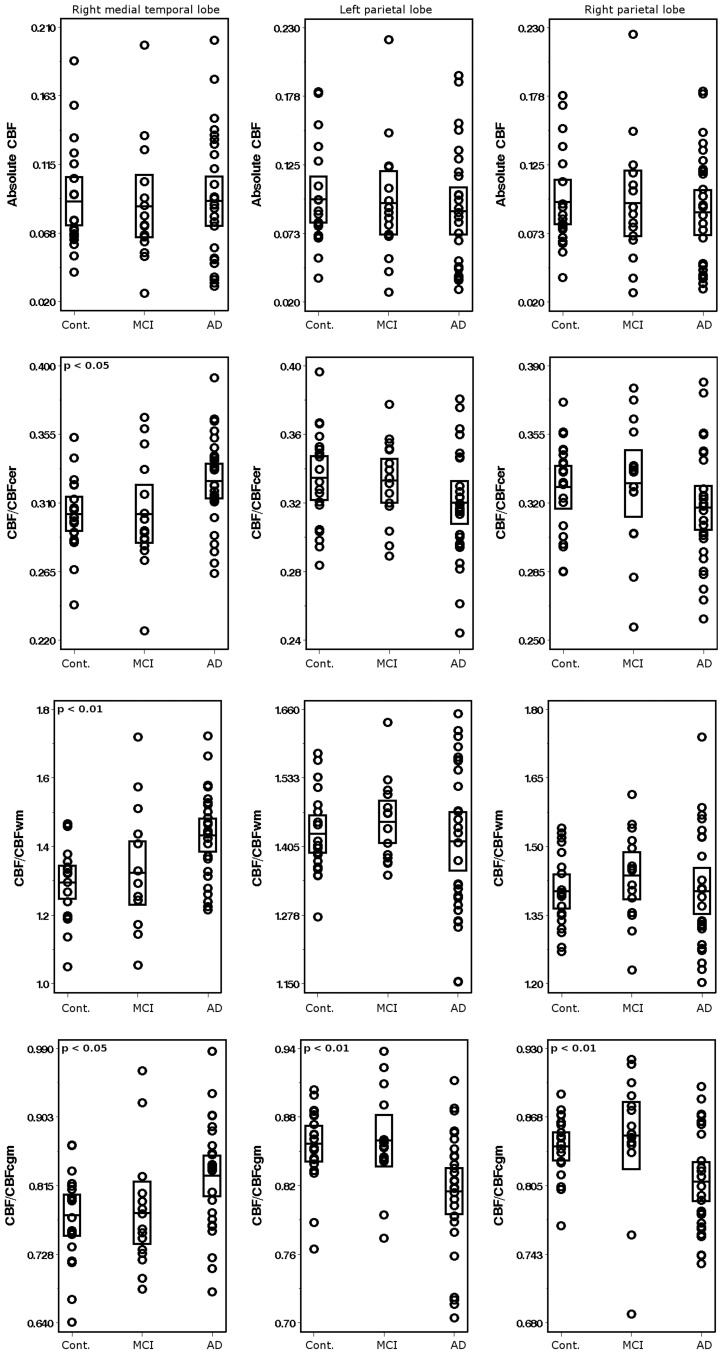
Scatter plots of CBF values for controls, patients with mild cognitive impairment, and patients with Alzheimer’s disease in both parietal lobes and right medial temporal lobe. Absolute CBF units are given in ml of blood/100 g of tissue/min. Cont, controls; MCI, mild cognitive impairment; AD, Alzheimer’s disease. Bar shows two standard deviations below and above the mean (horizontal line). *p*, one-way ANOVA *p* value.

No statistically significant differences between patient groups and controls were detected for absolute mean CBF values in cortical ROIs. Differences in perfusion values between groups were detected only after normalization of data. CBF data normalized by whole-brain cortical GM showed significantly lower CBF mean values in the right and left parietal lobes and higher mean values in the right medial temporal lobe in AD patients than in the control group (Fig. 2). No significant differences were found for the parietal lobes in AD patients according to whether the cerebellum or whole-brain WM was used as a reference region. The hyperperfusion pattern in the right medial temporal lobe appeared after normalization regardless of the reference region used; however, statistical significance was higher when normalization was by whole-brain WM than by the cerebellum or whole-brain cortical GM (Table 4). We found no perfusion differences between the group with stable MCI and controls either before or after normalization. Consistent results were found when the comparative analysis was performed based on cerebral gyri-based ROIs instead of lobe-based ROIs. Compared to controls, AD patients showed higher mean CBF values in the right fusiform gyrus (p<0.01) and right parahippocampal gyrus (p<0.05) after normalization by the three reference regions. This group also showed lower mean CBF values in the superior parietal gyri (right, p<0.05; left, p<0.01) and the right precuneus (p<0.05) after normalization by whole-brain cortical gray matter. Differences in the left superior parietal gyrus were also found after normalization by cerebellum, although the statistical significance was weaker (p<0.05). No significant differences were observed between the MCI group and controls.

**Table 4:** Dunnett’s test *p* values (*p*) and effect size (*d*) of differences between AD patients and controls in mean CBF value for the three reference regions studied.

	*Right temporal lobe (medial region)*		*Right parietal lobe*		*Left parietal lobe*	
	*p*	*d*	*p*	*d*	*p*	*d*
CBFcer	<0.05	0.76	–	–	–	–
CBFwm	<0.01	1.06	–	–	–	–
CBFcgm	<0.05	0.76	<0.05	−0.70	<0.01	−1.17

CBFcgm, cerebral blood flow of whole-brain cortical gray matter; CBFcer, cerebral blood flow of cerebellum; CBFwm, cerebral blood flow of whole-brain white matter; *p*, Dunnett’s test *p* value, *d*, Cohen’s coefficient.

### Reduction in Intra-group Variability after Normalization

Intra-group variability in the CBF measurements was considerably reduced after data normalization. Data normalized by whole-brain cortical GM consistently showed the lowest dispersion in every lobe (Table 5). The results of the component of variance analysis showed that the variance attributable to the normalization method was considerably larger (over 95%) than the variance attributable to the diagnosis group effect.

**Table 5:** Reduced variability in intensity values after normalization by the three reference regions (coefficients of variation, %).

	*Frontal*	*Parietal*	*Temporal*(lateral)	*Temporal*(medial)	*Occipital*
Absolute CBF	46.61	46.96	46.31	45.54	44.47
CBF/CBFcer	8.69	8.76	8.62	10.37	8.72
CBF/CBFwm	7.39	7.40	7.17	11.18	9.82
CBF/CBFcgm	5.54	5.40	5.94	7.98	5.73

CBF, cerebral blood flow; CBF/CBFcgm, CBF values per region of interest normalized by whole-brain cortical gray matter; CBF/CBFcer, CBF values per region of interest normalized by cerebellum; CBF/CBFwm, CBF values per region of interest normalized by whole-brain white matter. Coefficients of variation show bilateral information per lobe in the three groups (controls, MCI and AD).

### Medial Temporal Lobe Atrophy

Compared to controls, the AD group showed significantly lower GM volumes in the medial temporal cortex (bilateral), amygdalae and hippocampi. No significant differences in those regions were observed for the MCI group (table 6).

**Table 6:** Dunnett’s test *p* values for differences between patients and controls in gray matter volume for the medial temporal lobes.

	*Controls-MCI*	*Controls-Alzheimer*
Left temporal cortex	−	0.002
Right temporal cortex	−	<0.001
Left hippocampus	−	<0.0001
Right hippocampus	−	<0.0001
Left amygdala	−	<0.0001
Right amygdala	−	<0.0001

(−) non-significant differences.

## Discussion

The high physiological variability of CBF parametric maps makes it difficult to detect subtle perfusion abnormalities in AD, especially in small samples. Normalization of data based on a reference region usually facilitates the detection of disease-dependent affected regions. However, in comparative analyses, results could vary with the region chosen for normalization. This observation was reported in SPECT studies using 99mTc-hexamethylpropyleneamine oxime (HMPAO), which confirm the cerebellum to be the optimal region for normalization [11], [12], [13]; however, no studies to date have validated the cerebellum as the optimum normalization region in MRI perfusion studies. The present study shows the effect of using different regions to normalize the intensity of CBF parametric maps acquired by MRI on the results of a comparative analysis between stable MCI patients, AD patients and healthy controls. While SPECT studies confirmed the cerebellum as a valid reference region, our results suggest that it is not optimal in MRI studies in early-stage disease.

### Between-group Bias in Reference Regions

A possible disease-associated decrease in absolute CBF values must be ruled out before choosing a region as a reference. The three reference regions chosen for this study did not show any group-related bias. No between-group differences in mean values for absolute CBF data were found in the ANOVA model, and intra-group variability for CBF values was quite similar in all three regions (table 3). An insufficient sample size could be the reason for the undetected differences in global perfusion between patients and controls; in such cases, the criteria for accepting regions as unbiased is a *t* value close to zero [10]. In our case, since we compared three groups, we considered an *F* value close to zero as the criterion for an optimal normalization region. Whole-brain cortical GM showed the *F* value to be closest to zero (table 3).

### Effects of Normalization on between-group Analysis

Differences in perfusion values between groups were detected only after normalization of CBF data. We observed a statistically significant pattern of hyperperfusion in the right medial temporal lobe of AD patients after normalization. Relatively increased regional CBF in AD patients has been reported for SPECT and is commonly attributed to a reference region that is unsuitable for normalization. Such is the case of Soonawala et al. [13], who reported increased CBF in the occipital cortex and whole-brain WM using global normalization instead of the cerebellum. Talbot el al. [11] reported the same effect in the frontal lobe when they normalized by the occipital cortex instead of the cerebellum. However, the hyperperfusion pattern observed in the medial temporal lobe in AD patients in our study seems to be independent of the chosen reference region, since the same pattern appeared after normalization by the three regions studied. Increased CBF may suggest compensatory mechanisms [42] or inflammatory processes. Studies of inflammation in AD have reported activated microglia surrounding amyloid deposits within the brain, which may be responsible for a locally induced chronic inflammatory response [43], [44].

We also found a significant decrease in CBF in both parietal lobes in the AD group, although only when normalizing by whole-brain cortical GM. Cerebellum-normalized data showed the same hypoperfusion pattern, but group differences did not reach the threshold of statistical significance (Fig. 2). Similar hypoperfusion patterns in posterior regions of the parietotemporal cortex have been found in SPECT studies [45]. According to the disconnection hypothesis [46], the loss of synaptic inputs from the medial temporal lobe in the parietotemporal cortex reduces neuronal activity and blood flow in the region. This may be the reason why perfusion decreases in very early stages of the disease, when the region has not yet been affected by amyloid plaques [47]. The marked GM volume loss in the medial temporal lobes and the decreased parietal perfusion observed after normalization by whole-brain cortical GM are consistent with the pattern expected for early AD. A similar pattern of hyperperfusion in medial temporal lobes and hypoperfusion in parietal lobes has been described in a CBF study based on arterial spin labeling (ASL) MRI and normalization by the visual cortex instead of the cerebellum [19]. This study was based on a sample that was similar to ours in size, MMSE score and age. Although ASL presents methodological differences with respect to DSC (absence of external contrast and longer scan acquisition times, which make ASL more sensitive to patient movement artifacts [48]), measurements of perfusion by ASL seem to correlate well with DSC perfusion [49].

Nevertheless, other MRI studies have reported decreased parietal perfusion when the reference region was the cerebellum [16], [17]. However, the results may not be comparable to ours, because AD patients were older, with lower MMSE scores, and the variable assessed was cerebral blood volume instead of CBF, which seems to be more sensitive to physiological processes than cerebral blood volume [50].

According to our results, normalization by whole-brain cortical GM enables more sensitive detection of perfusion abnormalities (with a larger number of affected regions in AD patients) than data normalized by cerebellum or whole-brain WM.

We found no differences in perfusion between stable MCI patients and healthy controls before or after normalization. These findings may indicate vascular impairment that is specific to AD and not observed in MCI patients who do not progress to AD. In fact, in the MCI group, we did not observe GM volume loss in the medial temporal lobe, the first region affected by neuropathology in AD. The lack of evidence of AD in this group seems to indicate fulfillment of the specific criteria for MCI according to the new lexicon for Alzheimer’s disease proposed by Dubois et al. [51].

### Reduction in Intra-group Variability after Normalization

Absolute CBF data dispersion was considerably reduced after normalization by the three reference regions studied. However, normalization by whole-brain cortical GM showed the lowest coefficients of variation in all brain lobes (table 5). The reduced variability in CBF intensity values after normalization could arise from a reduction in intra-group variability, but also from a reduction in between-group differences. The component of variance analysis reveals that reduced variability was due to the first factor. This result could explain why more significant differences were observed between patients and controls after normalization of data. Since intensity variability across subject is the main obstacle to comparative analysis between controls and patients, the greater reduction in variability actually validates whole-brain cortical GM as a good reference region for MRI perfusion studies. One possible explanation is that cortical GM may be subject to sources of variability that are similar to those of the ROIs we studied; therefore, normalization against whole-brain cortical GM could somehow adjust for these hidden factors.

Our study is subject to a series of limitations. Given the differences in spatial resolution between T1 and PWI studies, coregistration may lead to tissue registration mismatch; therefore, some errors in cortical parcellation of CBF maps cannot be controlled. We accepted the results after normalization by whole-brain cortical GM as a more valid perfusion pattern than that obtained after normalizing by cerebellum and whole-brain WM, since similar temporoparietal hypoperfusion patterns have been demonstrated in SPECT studies where AD was confirmed by autopsy [52]. The main strengths of our study are the multimodal nature of the data, the application of an easily reproducible method for cortical brain parcellation and tissue segmentation, and the fact that patients with AD were recruited at a very early stage of the disease, since more than 50% of the patients had prodromal AD [51].

## Conclusion

Our results suggest that normalization of CBF parametric maps based on whole-brain cortical GM leads to a more sensitive detection of perfusion changes in AD patients than when the cerebellum or whole-brain WM is used as the reference region; therefore, the cerebellum is not necessarily the optimal reference region in MRI studies for early-stage AD. When normalizing by whole-brain cortical GM, we found a significant decrease in CBF in both parietal lobes and an increase in CBF in the right medial temporal lobe. This hyperperfusion pattern in the AD group seems to be independent of the chosen reference region. We found no differences in perfusion between the group with stable MCI and healthy controls either before or after normalization, thus suggesting the absence of perfusion abnormalities in this group.

## References

[1] Henry-Feugeas MC (2009) Assessing cerebrovascular contribution to late dementia of the Alzheimer’s type: the role of combined hemodynamic and structural MR analysis. J Neurol Sci 283: 44–48. 19268312 10.1016/j.jns.2009.02.325

[2] Zlokovic BV (2011) Neurovascular pathways to neurodegeneration in Alzheimer’s disease and other disorders. Nat Rev Neurosci 12: 723–738. 22048062 10.1038/nrn3114 PMC4036520

[3] Nagata K, Kondoh Y, Atchison R, Sato M, Satoh Y, et al (2000) Vascular and metabolic reserve in Alzheimer’s disease. Neurobiol Aging 21: 301–307. 10867215 10.1016/s0197-4580(00)00130-5

[4] Lecrux C, Hamel E (2011) The neurovascular unit in brain function and disease. Acta Physiol (Oxf) 203: 47–59. 21272266 10.1111/j.1748-1716.2011.02256.x

[5] Paulson OB, Hasselbalch SG, Rostrup E, Knudsen GM, Pelligrino D (2010) Cerebral blood flow response to functional activation. J Cereb Blood Flow Metab 30: 2–14. 19738630 10.1038/jcbfm.2009.188 PMC2872188

[6] Gonzalez RG, Fischman AJ, Guimaraes AR, Carr CA, Stern CE, et al (1995) Functional MR in the evaluation of dementia: correlation of abnormal dynamic cerebral blood volume measurements with changes in cerebral metabolism on positron emission tomography with fludeoxyglucose F 18. AJNR Am J Neuroradiol 16: 1763–1770. 8693972 PMC8338228

[7] Hanyu H, Sato T, Hirao K, Kanetaka H, Iwamoto T, et al (2010) The progression of cognitive deterioration and regional cerebral blood flow patterns in Alzheimer’s disease: a longitudinal SPECT study. J Neurol Sci 290: 96–101. 19931870 10.1016/j.jns.2009.10.022

[8] Petrella JR, Coleman RE, Doraiswamy PM (2003) Neuroimaging and early diagnosis of Alzheimer disease: a look to the future. Radiology 226: 315–336. 12563122 10.1148/radiol.2262011600

[9] Diamant M, Harms MP, Immink RV, Van Lieshout JJ, Van Montfrans GA (2002) Twenty-four-hour non-invasive monitoring of systemic haemodynamics and cerebral blood flow velocity in healthy humans. Acta Physiol Scand 175: 1–9. 11982498 10.1046/j.1365-201X.2002.00953.x

[10] Borghammer P, Jonsdottir KY, Cumming P, Ostergaard K, Vang K, et al (2008) Normalization in PET group comparison studies–the importance of a valid reference region. Neuroimage 40: 529–540. 18258457 10.1016/j.neuroimage.2007.12.057

[11] Talbot PR, Lloyd JJ, Snowden JS, Neary D, Testa HJ (1994) Choice of reference region in the quantification of single-photon emission tomography in primary degenerative dementia. Eur J Nucl Med 21: 503–508. 8082664 10.1007/BF00173036

[12] Karbe H, Kertesz A, Davis J, Kemp BJ, Prato FS, et al (1994) Quantification of functional deficit in Alzheimer’s disease using a computer-assisted mapping program for 99mTc-HMPAO SPECT. Neuroradiology 36: 1–6. 8107986 10.1007/BF00599183

[13] Soonawala D, Amin T, Ebmeier KP, Steele JD, Dougall NJ, et al (2002) Statistical parametric mapping of (99m)Tc-HMPAO-SPECT images for the diagnosis of Alzheimer’s disease: normalizing to cerebellar tracer uptake. Neuroimage 17: 1193–1202. 12414259 10.1006/nimg.2002.1259

[14] Ishii K, Sasaki M, Kitagaki H, Yamaji S, Sakamoto S, et al (1997) Reduction of cerebellar glucose metabolism in advanced Alzheimer’s disease. J Nucl Med 38: 925–928. 9189143

[15] Hauser T, Schonknecht P, Thomann PA, Gerigk L, Schroder J, et al (2013) Regional cerebral perfusion alterations in patients with mild cognitive impairment and Alzheimer disease using dynamic susceptibility contrast MRI. Acad Radiol 20: 705–711. 23664398 10.1016/j.acra.2013.01.020

[16] Bozzao A, Floris R, Baviera ME, Apruzzese A, Simonetti G (2001) Diffusion and perfusion MR imaging in cases of Alzheimer’s disease: correlations with cortical atrophy and lesion load. AJNR Am J Neuroradiol 22: 1030–1036. 11415893 PMC7974792

[17] Harris GJ, Lewis RF, Satlin A, English CD, Scott TM, et al (1998) Dynamic susceptibility contrast MR imaging of regional cerebral blood volume in Alzheimer disease: a promising alternative to nuclear medicine. AJNR Am J Neuroradiol 19: 1727–1732. 9802497 PMC8337487

[18] Uh J, Lewis-Amezcua K, Martin-Cook K, Cheng Y, Weiner M, et al (2009) Cerebral blood volume in Alzheimer’s disease and correlation with tissue structural integrity. Neurobiol Aging 31: 2038–2046. 19200623 10.1016/j.neurobiolaging.2008.12.010 PMC2888620

[19] Alsop DC, Casement M, de Bazelaire C, Fong T, Press DZ (2008) Hippocampal hyperperfusion in Alzheimer’s disease. Neuroimage 42: 1267–1274. 18602481 10.1016/j.neuroimage.2008.06.006 PMC2675915

[20] Alsop DC, Detre JA, Grossman M (2000) Assessment of cerebral blood flow in Alzheimer’s disease by spin-labeled magnetic resonance imaging. Ann Neurol 47: 93–100. 10632106

[21] Johnson NA, Jahng GH, Weiner MW, Miller BL, Chui HC, et al (2005) Pattern of cerebral hypoperfusion in Alzheimer disease and mild cognitive impairment measured with arterial spin-labeling MR imaging: initial experience. Radiology 234: 851–859. 15734937 10.1148/radiol.2343040197 PMC1851934

[22] Delis DC, Kramer JH, Kaplan E, Ober BA (2000) California Verbal Learning Test: Second Edition: Psychological, Corporation San Antonio, TX.

[23] Dubois B, Slachevsky A, Litvan I, Pillon B (2000) The FAB: a Frontal Assessment Battery at bedside. Neurology 55: 1621–1626. 11113214 10.1212/wnl.55.11.1621

[24] Lezak MD, Howieson DB, Loring DW (2004) Neuropsychological assessment: Oxford University Press, USA.

[25] Mathuranath PS, Nestor PJ, Berrios GE, Rakowicz W, Hodges JR (2000) A brief cognitive test battery to differentiate Alzheimer’s disease and frontotemporal dementia. Neurology 55: 1613–1620. 11113213 10.1212/01.wnl.0000434309.85312.19

[26] Spreen O, Strauss E (1998) A compendium of neuropsychological tests: Administration, norms, and commentary: Oxford University Press, USA.

[27] Winblad B, Palmer K, Kivipelto M, Jelic V, Fratiglioni L, et al (2004) Mild cognitive impairment–beyond controversies, towards a consensus: report of the International Working Group on Mild Cognitive Impairment. J Intern Med 256: 240–246. 15324367 10.1111/j.1365-2796.2004.01380.x

[28] Petersen RC, Smith GE, Waring SC, Ivnik RJ, Tangalos EG, et al (1999) Mild cognitive impairment: clinical characterization and outcome. Arch Neurol 56: 303–308. 10190820 10.1001/archneur.56.3.303

[29] American Psychiatric Association (2000) Diagnostic and statistical manual of mental disorders, 4th ed. text rev.: DSM-IV-TR. Washington, DC.

[30] Hughes CP, Berg L, Danziger WL, Coben LA, Martin RL (1982) A new clinical scale for the staging of dementia. Br J Psychiatry 140: 566–572. 7104545 10.1192/bjp.140.6.566

[31] McKhann G, Drachman D, Folstein M, Katzman R, Price D, et al (1984) Clinical diagnosis of Alzheimer’s disease: report of the NINCDS-ADRDA Work Group under the auspices of Department of Health and Human Services Task Force on Alzheimer’s Disease. Neurology 34: 939–944. 6610841 10.1212/wnl.34.7.939

[32] Folstein MF, Folstein SE, McHugh PR (1975) “Mini-mental state”. A practical method for grading the cognitive state of patients for the clinician. J Psychiatr Res 12: 189–198. 1202204 10.1016/0022-3956(75)90026-6

[33] Rempp KA, Brix G, Wenz F, Becker CR, Guckel F, et al (1994) Quantification of regional cerebral blood flow and volume with dynamic susceptibility contrast-enhanced MR imaging. Radiology 193: 637–641. 7972800 10.1148/radiology.193.3.7972800

[34] Li X, Tian J, Li E, Wang X, Dai J, et al (2003) Adaptive total linear least square method for quantification of mean transit time in brain perfusion MRI. Magn Reson Imaging 21: 503–510. 12878260 10.1016/s0730-725x(03)00075-4

[35] Fischl B, Dale AM (2000) Measuring the thickness of the human cerebral cortex from magnetic resonance images. Proc Natl Acad Sci U S A 97: 11050–11055. 10984517 10.1073/pnas.200033797 PMC27146

[36] Dale AM, Fischl B, Sereno MI (1999) Cortical surface-based analysis. I. Segmentation and surface reconstruction. Neuroimage 9: 179–194. 9931268 10.1006/nimg.1998.0395

[37] Rosas HD, Liu AK, Hersch S, Glessner M, Ferrante RJ, et al (2002) Regional and progressive thinning of the cortical ribbon in Huntington’s disease. Neurology 58: 695–701. 11889230 10.1212/wnl.58.5.695

[38] Kuperberg GR, Broome MR, McGuire PK, David AS, Eddy M, et al (2003) Regionally localized thinning of the cerebral cortex in schizophrenia. Arch Gen Psychiatry 60: 878–888. 12963669 10.1001/archpsyc.60.9.878

[39] Desikan RS, Segonne F, Fischl B, Quinn BT, Dickerson BC, et al (2006) An automated labeling system for subdividing the human cerebral cortex on MRI scans into gyral based regions of interest. Neuroimage 31: 968–980. 16530430 10.1016/j.neuroimage.2006.01.021

[40] Fischl B, Salat DH, Busa E, Albert M, Dieterich M, et al (2002) Whole brain segmentation: automated labeling of neuroanatomical structures in the human brain. Neuron 33: 341–355. 11832223 10.1016/s0896-6273(02)00569-x

[41] Collignon A, Maes F, Delaere D, Vandermeulen D, Suetens P, et al.. (1995) Automated multi-modality image registration based on information theory. Information Processing in Medical Imaging. Amsterdam: Kluwer Academic Publishers. pp. 263–274.

[42] Dickerson BC, Sperling RA (2008) Functional abnormalities of the medial temporal lobe memory system in mild cognitive impairment and Alzheimer’s disease: insights from functional MRI studies. Neuropsychologia 46: 1624–1635. 18206188 10.1016/j.neuropsychologia.2007.11.030 PMC2760288

[43] Salminen A, Ojala J, Kauppinen A, Kaarniranta K, Suuronen T (2009) Inflammation in Alzheimer’s disease: amyloid-beta oligomers trigger innate immunity defence via pattern recognition receptors. Prog Neurobiol 87: 181–194. 19388207 10.1016/j.pneurobio.2009.01.001

[44] Wilkinson BL, Cramer PE, Varvel NH, Reed-Geaghan E, Jiang Q, et al (2012) Ibuprofen attenuates oxidative damage through NOX2 inhibition in Alzheimer’s disease. Neurobiol Aging 33: 197.e121–e132. 10.1016/j.neurobiolaging.2010.06.014 PMC2980562 20696495

[45] Talbot PR, Lloyd JJ, Snowden JS, Neary D, Testa HJ (1998) A clinical role for 99mTc-HMPAO SPECT in the investigation of dementia? J Neurol Neurosurg Psychiatry 64: 306–313. 9527139 10.1136/jnnp.64.3.306 PMC2169991

[46] Jobst KA, Smith AD, Barker CS, Wear A, King EM, et al (1992) Association of atrophy of the medial temporal lobe with reduced blood flow in the posterior parietotemporal cortex in patients with a clinical and pathological diagnosis of Alzheimer’s disease. J Neurol Neurosurg Psychiatry 55: 190–194. 1564478 10.1136/jnnp.55.3.190 PMC1014723

[47] Braak H, Braak E (1991) Neuropathological stageing of Alzheimer-related changes. Acta Neuropathol 82: 239–259. 1759558 10.1007/BF00308809

[48] Essig M, Nguyen TB, Shiroishi MS, Saake M, Provenzale JM, et al (2013) Perfusion MRI: The Five Most Frequently Asked Clinical Questions. AJR Am J Roentgenol 201: W495–510. 23971482 10.2214/AJR.12.9544 PMC3842445

[49] Huang YC, Liu HL, Lee JD, Yang JT, Weng HH, et al (2013) Comparison of arterial spin labeling and dynamic susceptibility contrast perfusion MRI in patients with acute stroke. PLoS One 8: e69085. 23874876 10.1371/journal.pone.0069085 PMC3712946

[50] Grubb RL Jr, Raichle ME, Eichling JO, Ter-Pogossian MM (1974) The effects of changes in PaCO2 on cerebral blood volume, blood flow, and vascular mean transit time. Stroke 5: 630–639. 4472361 10.1161/01.str.5.5.630

[51] Dubois B, Feldman HH, Jacova C, Cummings JL, Dekosky ST, et al (2010) Revising the definition of Alzheimer’s disease: a new lexicon. Lancet Neurol 9: 1118–1127. 20934914 10.1016/S1474-4422(10)70223-4

[52] Jagust W, Thisted R, Devous MD Sr, Van Heertum R, Mayberg H, et al (2001) SPECT perfusion imaging in the diagnosis of Alzheimer’s disease: a clinical-pathologic study. Neurology 56: 950–956. 11294935 10.1212/wnl.56.7.950

